# Comprehensive and integrative analyses identify *TYW5* as a schizophrenia risk gene

**DOI:** 10.1186/s12916-022-02363-8

**Published:** 2022-05-09

**Authors:** Chengcheng Zhang, Xiaojing Li, Liansheng Zhao, Rong Liang, Wei Deng, Wanjun Guo, Qiang Wang, Xun Hu, Xiangdong Du, Pak Chung Sham, Xiongjian Luo, Tao Li

**Affiliations:** 1 Mental Health Center and Psychiatric Laboratory, the State Key Laboratory of Biotherapy, West China Hospital of Sichuan University, Chengdu, Sichuan China; 2 Department of Neurobiology, Affiliated Mental Health Center & Hangzhou Seventh People’s Hospital, Zhejiang University School of Medicine, Hangzhou, Zhejiang 310013 People’s Republic of China; 3 The Clinical Research Center and Department of Pathology, The Second Affiliated Hospital, Zhejiang University School of Medicine, Hangzhou, Zhejiang China; 4 Suzhou Psychiatric Hospital, The Affiliated Guangji Hospital of Soochow University, Suzhou, China; 5 Department of Psychiatry, Li Ka Shing Faculty of Medicine, The University of Hong Kong, Hong Kong, SAR China; 6 Centre for PanorOmic Sciences, The University of Hong Kong, Hong Kong, SAR China; 7 State Key Laboratory of Brain and Cognitive Sciences, The University of Hong Kong, Hong Kong, SAR China; 8 Kunming College of Life Science, University of Chinese Academy of Sciences, Kunming, Yunnan China; 9 NHC and CAMS Key Laboratory of Medical Neurobiology, MOE Frontier Science Center for Brain Science and Brain-machine Integration, School of Brain Science and Brain Medicine, Zhejiang University, Hangzhou, Zhejiang China

**Keywords:** Schizophrenia, GWAS, eQTL, MRI, *TYW5*

## Abstract

**Background:**

Identifying the causal genes at the risk loci and elucidating their roles in schizophrenia (SCZ) pathogenesis remain significant challenges. To explore risk variants associated with gene expression in the human brain and to identify genes whose expression change may contribute to the susceptibility of SCZ, here we report a comprehensive integrative study on SCZ.

**Methods:**

We systematically integrated the genetic associations from a large-scale SCZ GWAS (*N* = 56,418) and brain expression quantitative trait loci (eQTL) data (*N* = 175) using a Bayesian statistical framework (Sherlock) and Summary data-based Mendelian Randomization (SMR). We also measured brain structure of 86 first-episode antipsychotic-naive schizophrenia patients and 152 healthy controls with the structural MRI.

**Results:**

Both Sherlock (*P* = 3. 38 × 10^−6^) and SMR (*P* = 1. 90 × 10^−8^) analyses showed that *TYW5* mRNA expression was significantly associated with risk of SCZ. Brain-based studies also identified a significant association between TYW5 protein abundance and SCZ. The single-nucleotide polymorphism rs203772 showed significant association with SCZ and the risk allele is associated with higher transcriptional level of *TYW5* in the prefrontal cortex. We further found that *TYW5* was significantly upregulated in the brain tissues of SCZ cases compared with controls. In addition, *TYW5* expression was also significantly higher in neurons induced from pluripotent stem cells of schizophrenia cases compared with controls. Finally, combining analysis of genotyping and MRI data showed that rs203772 was significantly associated with gray matter volume of the right middle frontal gyrus and left precuneus.

**Conclusions:**

We confirmed that *TYW5* is a risk gene for SCZ. Our results provide useful information toward a better understanding of the genetic mechanism of *TYW5* in risk of SCZ.

**Supplementary Information:**

The online version contains supplementary material available at 10.1186/s12916-022-02363-8.

## Background

Schizophrenia (SCZ) is a severe, highly heritable and heterogeneous disease characterized by positive symptoms (e.g., hallucinations, delusions), negative symptoms (e.g., apathy, lack of emotion, poor social functioning), and cognitive deficits [1]. It affects approximately 0.5–1% of the world population and accounts for 1.1% of the total disability-adjusted life years [2]. Moreover, onset of SCZ typically manifests during late adolescent and early adulthood [3]. So far, the pathophysiology of SCZ has not been fully understood due to the phenotypic and psychopathological complexity and heterogeneity [4]. The neurodevelopmental model of schizophrenia posits that early neurodevelopmental abnormalities of brain development may have a role in SCZ [5–8]. According to the neurodevelopmental hypothesis, schizophrenia is mainly caused by genetic components, which affects prenatal and postnatal neurodevelopment. Environmental events increase the risk for phenotype expression of schizophrenia among these genetically susceptible individuals [5]. For example, fetal hypoxia was associated with significant decreases in gray matter density among schizophrenia patients and their healthy siblings, but not non-familial controls [9]. Moreover, substantial evidence has linked neurodevelopmental insults to a series of substantial risk genes for schizophrenia [10]. For example, the schizophrenia risk gene, *NRGN*, bidirectionally modulates synaptic plasticity via regulating the neuronal phosphoproteome [11]. Evidence points to the role of *DISC1* in regulation of intracellular trafficking of a wide range of neuronal cargoes [12]. Furthermore, the *C4A* expression and structural variation have been found associated with neurodevelopment in schizophrenia [13].

Given the importance of genetic influences on schizophrenia susceptibility, there are increasingly well-powered genomic studies on identifying disease-related variants and loci [14, 15]. For example, the Schizophrenia Working Group of the Psychiatric Genomics Consortium reported 108 independent risk loci based on a multi-stage genome-wide association study (GWAS) in ~150,000 individuals [16]. More recently, using GWAS, Lam and colleagues compiled the largest East Asian genetics cohort and identified 208 significant associations in 176 genetic loci between East Asian and European ancestries, suggesting consistency of schizophrenia risk alleles across ethnicities and cultures [17]. As accumulating genomic variations are found to be associated with SCZ, genetic risk factors likely explain the common clinical and etiological features of schizophrenia (e.g., brain expression changes and morphological impairment in gray matter) [18]. Therefore, identifying potential risk loci and elucidating how they affect schizophrenia pathogenesis will provide important knowledge about the pathophysiology of SCZ. On the other hand, approximately 90% of the single-nucleotide polymorphisms (SNPs) or variants identified by GWAS were located in a noncoding region [19]. One explanation is that noncoding variants may exert functional impacts through modulating mRNA expression of nearby or distal genes [20]. For example, through integrating expression quantitative trait loci (eQTL) and GWAS, Zhang and colleagues [21] showed that SCZ risk variant rs2071287 might confer SCZ risk by modulating *NOTCH4* expression. Consistently, *NOTCH4* was significantly downregulated in schizophrenia patients compared with controls [21]. Integrative approaches (such as Sherlock integrative analysis, a Bayesian approach that combining genetic associations from GWAS and human brain eQTL data) provide valuable insights into the gene regulatory mechanisms of schizophrenia [22]. For instance, a recent report identified *LRP8* as a schizophrenia risk gene by integrative analysis [23]. In addition, network analysis showed that LRP8 directly participates in a highly interconnected protein-protein interaction network built by top risk genes for SCZ [24].

Previous studies have linked tRNA modification and metabolic abnormalities to neurodevelopmental disorder [25, 26]. *TYW5* (tRNA-YW Synthesizing Protein 5) is a major tRNA hydroxylase involved in epigenetic modification in brain [27–29]. Some researchers have shown a link between *TYW5* and mental illnesses such schizophrenia [15, 30], whereas others have been unable to corroborate this association [31, 32]. More studies are needed to determine whether *TYW5* is a risk gene for schizophrenia. More importantly, little is known about how *TYW5* genetic variations confer schizophrenia susceptibility or the role of *TYW5* in schizophrenia pathophysiology. Here, we employed Sherlock and Summary data-based Mendelian Randomization (SMR) integrative analysis integrate disease associations and eQTL signatures in GWAS loci, to discover *TYW5* as a SCZ risk gene, which is likely to play a role in SCZ pathogenesis. We also examined the expression level of *TYW5* in the dorsolateral prefrontal cortex of schizophrenia cases and controls using expression data. Furthermore, we explored the potential role of *TYW5* in schizophrenia pathogenesis using induced pluripotent stem cells. Our findings support that *TYW5* is a schizophrenia risk gene whose expression may be regulated by schizophrenia GWAS SNPs. Finally, we also provided evidence for an association between *TYW5* and gray matter volume abnormalities in the frontal-partial regions, suggesting the potential pathophysiological role of *TYW5* in SCZ.

## Methods

### GWAS associations of schizophrenia

We used the GWAS study results (PGC EAS and EUR SCZ) conducted by Lam et al. in the current integrative study [17]. Briefly, Lam et al. [17] first conducted a genome-wide meta-analysis through using 22,778 schizophrenia cases and 35,362 controls. These samples were from 20 East Asia sites (IMH-1, HNK-1, JPN-1, IMH-2, BIX-1, BIX-2, BIX-3, BIX-5, XJU-1, BIX-4, UMC-1, SIX-1, UWA-1, BJM-1, BJM-2, BJM-3, BJM-4, TAI-1, TAI-2, and KOR-1). They then combined these samples (Stage 1 and Stage 2) with 33,640 cases and 43,456 controls from the PGC2 GWAS, which resulted in a total of 135,236 individuals (including 56,418 cases and 78,818 controls). Meta-analysis was performed as previously described [24] using PLINK (the fixed-effect model was used) [33]. More details about sample description, genotyping, and statistical analyses could be found in the original publication [17].

### Brain eQTL data

Previous studies suggested the frontal cortex, which is the hub of most higher cognitive functions in human brain, has important roles in SCZ [34]. We thus used the frontal cortex as a primary brain region to study the effects of SCZ risk variants on gene expression. In present study, we used brain expression quantitative trait loci (eQTL) from the GTEx consortium [35]. In brief, 449 individuals of European ancestry across 44 human tissues were collected by GTEx consortium. They created eQTL resources by integrating genotypes with gene expression (measured with polyA+ selected RNA-seq) using Matrix eQTL. The eQTL association results of the frontal cortex tissues (175 individuals) from GTEx (https://www.gtexportal.org/) were used in the current analysis. These results were derived through linear regression analyses and adjusted for top three genotyping principal components, gender, genotyping platforms, and additional covariates.

### Sherlock integration analysis of SCZ GWAS and brain eQTL

Sherlock (http://sherlock.ucsf.edu/submit.html) is a powerful integrative method that computes the Bayes factor for each gene [36]. By integrating the GWAS associations and the eQTL data from a related tissue, Sherlock can identify genes whose expression change may play a role in disease risk [36]. For a given gene, there may be many variants in the genome affecting its expression (expression SNPs, eSNPs). A change of genotype at any of these eSNPs would lead to mRNA expression change of the gene, which could, in turn, affect the disease risk. Sherlock computes the logarithm of Bayes factor (LBF) score for each eSNP of the gene being tested to reflect the association strength between SNP and disease. We set the P thresholds for *cis* and *trans* associations at 1.0 × 10^−3^ and 5.0 × 10^−5^, respectively (as recommended by the default settings of Sherlock). More detailed information about the principle of Sherlock, statistical model, and LBF calculation can be found in the paper by He and colleagues [36].

### SMR integrative analysis

We used the summary data-based Mendelian randomization (SMR), an independent integrative analysis approach developed by Zhu et al. to validate our findings [37]. Similar to Sherlock, SMR employs the Mendelian randomization to test for pleiotropic associations between gene expression and complex traits by using eQTL and trait GWAS summary data. Only SNP-gene associations with *P* values < 5 × 10^−8^ (--peqtl-smr 5.0e-8) were included in the SMR analysis. The SMR details about the SMR analyses can be found in the original paper [37].

### SMR integrative analysis using protein quantitative trait loci

Considering that protein is the most common drug target and disease indicator, in addition to performing SMR using eQTL, we also performed SMR using SNP-protein abundance weights (i.e., protein quantitative trait loci, pQTL), to test whether SCZ risk genes (from the Sherlock approach) were associated with SCZ via their cis-regulated brain protein abundance. The pQTL data in the dorsolateral prefrontal cortex (DLPFC) of 376 people from the Religious Orders Study/Memory and Aging Project (ROS/MAP) and in the DLPFC of 152 people from Banner Sun Health Research Institute (Banner) proteomic, as well as SCZ GWAS summary statistics, were utilized to conduct SMR [38].

### Brain expression analysis of TYW5 in SCZ

Sherlock identifies SCZ-associated genes under the assumption that gene expression change may have a role in the pathogenesis of SCZ. To further explore if the genes identified by Sherlock integrative analysis were dysregulated in patients with SCZ, we compared the brain expression level of the genes identified by Sherlock in cases with SCZ and controls using the expression data from the CommonMind Consortium data. We downloaded the normalized DLPFC gene expression profile of schizophrenia and controls from Synapse (https://www.synapse.org, syn5609493) [22]. This dataset contained mRNA expression data of the postmortem human brain specimens from three brain banks (the Icahn School of Medicine at Mount Sinai, the University of Pennsylvania, and the University of Pittsburg). Briefly, the total RNA was isolated from 258 schizophrenia cases and 279 healthy controls. The sequencing was performed using Illumina HiSeq 2500 after quality control by RNA integrity number. The cleaned sequencing reads were mapped to human reference genome hg19 (http://www.ensembl.org/info/data/ftp/index.html), and gene expression levels were quantified using log (CPM) (read counts per million total reads).

### Analysis of TYW5 expression in neurons induced from iPSCs of SCZ cases and controls

It has become apparent that induced pluripotent stem cells (iPSCs) provide a pivotal opportunity to study the stepwise differentiation of patient-derived cells into neurons to identify alterations in cellular behavior and test novel therapeutic approaches. The expression of *TYW5* in iPSCs and neurons derived from SCZ patients and healthy controls has been analyzed [39]. In brief, subjects diagnosed with SCZ or healthy controls contributing a skin sample (healthy controls were ascertained through the laboratories of B. Cohen (McLean Hospital), D. Weinberger (Lieber Institute for Brain Development), and J. Rapoport (National Institute of Mental Health)), which was used for iPSC generation. To characterize the iPSCs, as well as to determine whether there were differences in their gene expression profiles with cortical interneurons differentiation, total RNA was isolated from 24 individual iPSC cell lines (4 SCZ lines and 4 controls lines, each line with 3 independent differentiations) after 8 weeks of neuronal differentiation, using the TRIzol reagent (Invitrogen, Grand Island, NY, USA). Stranded cDNA libraries were prepared using a TruSeq Stranded H.T. or L.T. mRNA kit (Illumina) following the manufacturer’s protocol using polyadenylated RNA isolation. Two-tailed Student’s *t* test was used to compare if the difference was significant (*P* < 0.05). Detailed protocols about fibroblast derivation, iPSC derivation, and neuronal differentiation were described previously [39].

### TYW5 expression analysis in multiple human tissues

We used gene expression data from the GTEx (Genotype-Tissue Expression, v7p release) to investigate *TYW5* gene expression profiling in various human tissues. We downloaded data from 53 human tissues for gene expression (i.e., median values of gene expression) [35]. The gene expression was quantified using RNA sequencing (reads per kilobase million (RPKM) to represent each gene’s expression level). More details can be found on GTEx (http://gtexportal.org/) and the associated publications [35] (https://gtexportal.org/home/publicationPage) on tissue selection, RNA extraction, expression quantification, and data processing.

### TYW5 expression in multiple central nervous system cell types

Zhang et al. isolated cell types of cells in human brains including microglia, astrocytes, oligodendercytes, endothelial cells, and neurons and conducted transcriptomal analysis using the RNA sequencing method [40]. We compared the expression levels of *TYW5*, extracted as fragments per kilobase of transcript per million mapped reads (FPKM), in different cell types. We also investigated *TYW5* expression in different mouse-brain cell types using Cahoy et al. data in multiple cell types of the mouse brain to compare whether *TYW5* displays a similar expression pattern [41].

### Temporal and spatial expression patterns of TYW5 in the developing and adult brain

To investigate the possible role of *TYW5* in the central nervous system, we investigated the temporal expression pattern of *TYW5* in the developing and adult human brain. We used two different expression data sets in this analysis. The first expression dataset was from the BrainSpan (Atlas of the Evolving Human Brain) (http://www.brainspan.org/). Gene expression values (based on RNA sequencing) of *TYW5* in the prefrontal cortex (PFC) (*N* = 42) were downloaded and transformed as described previously [42]. We used the transformed expression level to plot the temporal expression pattern of *TYW5* in developing and adult human brains. The second expression dataset was from the study of PsychENCODE (http://development.psychencode.org) [43]. The PsychENCODE generated transcriptomic profiling data (mRNA-seq) of 607 histologically verified, high-quality tissue samples from 16 anatomical brain regions dissected from 41 brains with an age range from 8 postconceptional weeks to 40 postnatal years. Detailed information about sample origin, sample size, and data processing can be found in the original publication [43].

### Association analysis between SNPs in TYW5 and the expression level of TYW5

We analyzed the associations between the risk SNPs in *TYW5* and gene expression levels to detect the potential functional effects of the risk variants in *TYW5*. We utilized the DLPFC eQTL datasets from BrainSeq Phase 1 (*n* = 412). The BrainSeq Phase 1 contains PolyA^+^ RNA-seq results obtained from human DLPFC tissues of 412 subjects (175 schizophrenia patients and 237 unaffected controls, age > 13). The eQTL associations were analyzed using linear regression under an additive genetic effect model and adjusted for sex, ancestry, and expression heterogeneity (principal components). From the BrainSeq Phase 2, a RiboZero RNA-seq eQTL dataset of human brain tissues [44], we retrieved data of the DLPFC from 397 individuals aged 13 or older. MatrixEQTL [45] was used to identify eQTL for gene-level expression based on the formula: log_2_(RPKM + 1) ~ SNP + diagnosis + sex + SNP PCs + expression PCs. Notably, the samples in BrainSeq Phase 1 and 2 were partially overlapped; however, both datasets were still included considering the different RNA-seq methods.

### Association analysis between SNPs in TYW5 and the brain gray matter volume

#### Subjects

To analyze the associations between the risk SNPs in *TYW5* and whole-brain gray matter volume, we recruited 86 patients with schizophrenia and 152 healthy controls from the West China Hospital of Sichuan University. We interviewed all participants using the Structured Clinical Interview for Diagnostic and the Statistical Manual of Mental Disorders, Fourth Edition, Text Revision (DSM- IV-TR) Axis I Disorders (SCID, patient edition and non-patient edition). The patients with schizophrenia were first-episode, drug-naïve at the time of evaluation. The inclusion and exclusion criteria are as follows: (1) aged between 16 and 50; (2) Han Chinese; (3) right-handed; (4) experience the first episode of schizophrenia and treatment naïve or ≤ 3 days of antipsychotic treatment before clinical assessment and MRI scan; (5) fulfill the diagnosis criteria of schizophrenia in the Diagnostic and Statistical Manual of Mental Disorders (DSM-IV); (6) I.Q. ≥ 70 according to Wechsler IQ Test; (7) the current episode cannot be accounted for by any specific life events.

#### Genotyping

DNA was extracted from the peripheral blood of each participant following the standard phenol-chloroform protocol. rs203772 genotyping was performed using Infinium Global Screening Array-24 v1.0 BeadChip. Quality control was performed as follows: SNPs were filtered based on unmatched gender information (between genomic sex and self-reported gender), missing genotype rate (≥ 3%), heterozygosity (≥ 3rd standard deviations), Hardy–Weinberg equilibrium (*P* < 0.001) and minor allele frequency (MAF < 0.01). The Sanger Imputation Server (https://imputation.sanger.ac.uk/) was used with SHAPEIT for phasing [46]. The positional Burrows-Wheeler transform was used for imputation and the Haplotype Reference Consortium v1.1 was used as the reference panel. Quality control procedures were then conducted to remove subjects with high missing genotype rate (≥ 3%) and exclude SNPs with poor imputation quality (INFO <0.3) and MAF <0.01. In order to further guarantee the validity of genotypes of rs203772, we then genotyped rs203772 using Sanger sequencing according to the manufacturer’s protocol. Finally, genotypes of 86 patients with first-episode treatment-naive SCZ and 152 healthy controls were analyzed.

The SNPs’ allele frequencies were in the Hardy–Weinberg equilibrium in healthy controls (79 GG, 57 AG, 16 AA, *P* = 0.24) and patients with SCZ (47 GG, 33 AG, 6 AA, *P* = 0.99). Age, handedness, sex, and level of education did not significantly differ between patients and control groups. A comparative profile of rs203772 genotype distribution between first-episode untreated SCZ patients and controls was shown in Additional file 1: Table S1.

#### Magnetic resonance images (MRI) scanning

MRI were acquired using a Philips 3T (Achieva, TX, Best, the Netherlands) scanner using an eight-channel head coil. Participants underwent a high resolution 3-dimensional T1-weighted, sagittal, magnetization-prepared rapid gradient echo (MPRAGE) sequence with the following parameters: repetition time (TR) = 8.37 ms, echo time (TE) = 3.88 ms, flip angle = 7°, in-plane matrix resolution = 256 × 256, FOV(field of view) = 24 cm × 24 cm, voxel size = 1 × 1 × 1mm^3^, thickness =1 mm with no gap, number of slices = 188.

#### Structural imaging processing

We processed the structural images with a MatLab toolbox statistical parametric mapping 12 (SPM12, http://www.fil.ion.ucl.ac.uk/spm). We used Diffeomorphic Anatomical Registration Through Exponentiated Lie algebra (DARTEL) toolbox to perform voxel-based morphometry (VBM) analysis with default parameters. All images were then normalized to the standard Montreal Neurological Institute (MNI) template, modulated to account for volume changes in the warping, and resampled to 1.5 × 1.5 × 1.5 mm^3^. Modulated gray matter images were smoothed with a 10-mm Gaussian kernel. An explicit mask was used from the SPM intracranial brain template to restrict which voxels should undergo statistical analysis. A significant difference was set as a voxel-wise threshold of *P* < 0.001 [47, 48]. The general linear model was used to test interaction effect of genotype (the G/G- carriers versus the G/A and A/A carriers) and diagnosis (SCZ versus controls) on brain morphology by using a voxel-by-voxel statistical analysis, controlling for the potential confounding effects of age, sex, and total brain volume.

## Results

### Integrative analyses of brain eQTL data and GWAS prioritized high-confidence risk genes for SCZ

The overall study design and relevant rationale were shown in Additional file 1: Figure S1. Based on the GWAS statistics of 56,418 SCZ patients and 78,818 controls [24], we conducted multi- SNP-based Sherlock to examine the associations between gene expression (frontal eQTL datasets from GTEx dataset [35]) and SCZ risk. We identified multiple genes whose mRNA expression levels may significantly affect the risk of SCZ at *FDR adjusted P*-value <0.01 (Table 1). Notably, some of these genes have been previously reported in studies of psychiatric illnesses, such as *GLT8D1* [49], *BTN3A2* [50], *FTCDNL1* [51], and *C4A* [13]. For each gene, at least one SNP showed significant association with SCZ and the expression of this gene simultaneously, suggesting that these genetic variants may confer SCZ risk by affecting these genes’ expression. The SCZ risk SNPs that showed the most significant association with gene expression are rs3130614 (associated with expression of *C4A*, eQTL *P* = 5.65 × 10^−19^), rs11694369 (associated with expression of *FTCDNL1*, eQTL *P* = 8.15 × 10^−16^) and rs203772 (associated with expression of *TYW5*, eQTL *P* = 2.74 × 10^−10^).

**Table 1:** Top SCZ-associated genes predicted by Sherlock integrative analysis

Gene	CHR	BP^a^	LBF^b^	*P* value^c^	Supporting SNP	Cis or Trans	GWAS *P* value^d^	eQTL *P* value^e^	FDR^f^
CYP21A1P	6	32146528	15.0977	3.38E-06	rs1150755	cis	1.30E-07	6.36E-09	<0.01
					rs2854275	cis	2.26E-08	1.13E-10	
C4A	6	31584437	14.9444	3.38E-06	rs3130614	cis	1.28E-08	5.65E-19	<0.01
					rs2854275	cis	2.41E-06	1.13E-10	
CSPG4P12	15	82898038	7.55567	3.38E-06	rs11635505	cis	2.57E-07	4.95E-13	<0.01
BAG6	6	31834667	7.55201	3.38E-06	rs707939	cis	4.57E-08	4.70E-13	<0.01
GLT8D1	3	52794367	7.4461	3.38E-06	rs2268023	cis	3.42E-06	3.66E-09	<0.01
PCDHA10	5	140138726	7.40982	3.38E-06	rs4451093	cis	1.28E-13	1.20E-10	<0.01
GNL3	3	52597126	7.39453	3.38E-06	rs2590838	cis	4.54E-06	8.82E-13	<0.01
**TYW5**	2	200598933	7.37493	3.38E-06	rs203772	cis	1.83E-08	2.74E-10	<0.01
ITIH4	3	52849336	7.35272	3.38E-06	rs6445539	cis	6.21E-09	1.17E-09	<0.01
DDAH2	6	31837338	7.34818	3.38E-06	rs707938	cis	6.04E-06	1.07E-11	<0.01
DNM1P51	15	82549305	7.3218	3.38E-06	rs12915234	cis	9.24E-13	4.34E-13	<0.01
DDHD2	8	38215456	7.30636	3.38E-06	rs6992943	cis	8.37E-07	8.02E-12	<0.01
GOLGA2P7	15	82722026	7.29214	3.38E-06	rs12906983	cis	7.03E-12	5.63E-12	<0.01
BTN3A2	6	26485364	7.17497	3.38E-06	rs9366655	cis	3.34E-14	1.34E-14	<0.01
CORO7	16	4424614	7.16594	3.38E-06	rs4785964	cis	7.76E-08	4.03E-08	<0.01
FTSJ2	7	2242519	7.11154	3.38E-06	rs7787274	cis	7.63E-07	9.36E-08	<0.01
HLA-J	6	29753592	6.93651	3.38E-06	rs3129063	cis	8.86E-06	1.70E-06	<0.01
FTCDNL1	2	200449965	6.92637	3.38E-06	rs11694369	cis	1.47E-07	8.15E-16	<0.01
ZSCAN23	6	28496744	6.84615	3.38E-06	rs2531831	cis	2.02E-06	1.78E-08	<0.01
MAPK3	16	29854171	6.73467	3.38E-06	rs11649612	cis	3.70E-06	5.00E-10	<0.01
PCDHA9	5	140228723	6.60836	3.38E-06	rs192231	cis	3.29E-07	1.10E-08	<0.01
NDUFAF7	2	37393080	6.60052	3.38E-06	rs10190959	cis	1.56E-06	1.86E-07	<0.01
ZNF204P	6	27420057	6.58274	3.38E-06	rs764284	cis	3.38E-07	1.12E-11	<0.01

TYW5 was shown in bold

^a^gene locations were based on hg19

^b^*LBF* logarithm of Bayes factor. The LBF score of a gene reflects the association strength between this gene and SCZ. For example, a LBF of 5.0 means that the gene is more likely to be associated with the disease (exp(5.0) = ~148 times) than no association

^c^*P* value from Sherlock integrative analysis. Larger LBF corresponds to smaller *P* value

^d^GWAS P value indicates the association significance between this SNP and SCZ

^e^eQTL *P* value indicates the association significance between this SNP and gene expression in brain

^f^FDR was corrected by Benjamini–Hochberg procedure

### Validation of the SCZ risk genes using SMR

In addition to *Sherlock*, we also conducted the *SMR* analysis to examine whether schizophrenia risk genes can be verified by integrating brain eQTL [35] and schizophrenia GWAS from Lam et al. [17]. Consistent with *Sherlock* results, using independent integrative analytic techniques, we provided further evidence to verify 11 genes, including *TYW5* (Top SNP rs203772, *P*_*SMR*_=1.90 × 10^−8^), as authentic risk genes for SCZ (Table 2).

**Table 2:** Validation of schizophrenia risk genes using independent integrative analysis method (SMR)

Gene symbol	CHR	Top SNP	*P*_GWAS_	*P*_eQTL_^a^	*P*_SMR_^b^	Corrected *P*_SMR_
TYW5	2	rs203772	1.83E-08	2.74E-10	1.90E-08	<0.05
CYP21A1P	6	rs2854275	2.26E-08	1.13E-10	2.60E-08	<0.05
C4A	6	rs1150752	4.05E-08	1.94E-17	4.55E-08	<0.05
BAG6	6	rs707939	4.57E-08	4.70E-13	4.72E-08	<0.05
PCDHA10	5	rs2240694	7.30E-14	3.05E-10	8.32E-14	<0.05
ITIH4	3	rs6445539	6.21E-09	1.17E-09	6.59E-09	<0.05
DNM1P51	15	rs12911210	9.24E-13	4.46E-13	1.07E-12	<0.05
BTN3A2	6	rs1977	3.34E-14	5.30E-10	4.31E-14	<0.05
FTCDNL1	2	rs13008446	8.04E-19	6.68E-01	1.07E-18	<0.05
MAPK3	16	rs2005219	1.20E-09	9.61E-05	1.29E-09	<0.05
PCDHA9	5	rs155799	1.90E-10	1.40E-04	2.06E-10	<0.05

^a,b^SMR integrative analysis was performed using schizophrenia GWAS from the PGC and brain eQTL from GTEx

Furthermore, we conducted the *SMR* analysis to examine whether schizophrenia risk genes can be verified by integrating brain pQTL [38] and schizophrenia GWAS from Lam et al. [17]. We provided further evidence that showed that *TYW5* (discovery *P*_*SMR*_=4.58 × 10^−5^ in ROS/MAP proteomic profiles and replication *P*_*SMR*_=5.37 × 10^−4^ in Banner proteomic profiles) is a risk gene for SCZ, which was consistent with *Sherlock* results (Table 3).

**Table 3:** Validation of schizophrenia risk genes using integrative analysis method (SMR) with pQTL datasets

Gene	CHR	*P*_SMR_^a^	*P*_SMR_^b^
TYW5	2	4.58E-05	5.37E-04
ITIH4	3	8.32E-03	-
MAPK3	16	4.96E-04	2.82E-02

^a^ SMR integrative analysis was performed using schizophrenia GWAS from the PGC and brain pQTL from ROSMAP. ^b^ SMR integrative analysis was performed using schizophrenia GWAS from the PGC and brain pQTL from Banner. - Protein not profiled in the proteomic dataset

### Upregulation of TYW5 in brain and iPSC neurons in SCZ

**Fig. 1:**
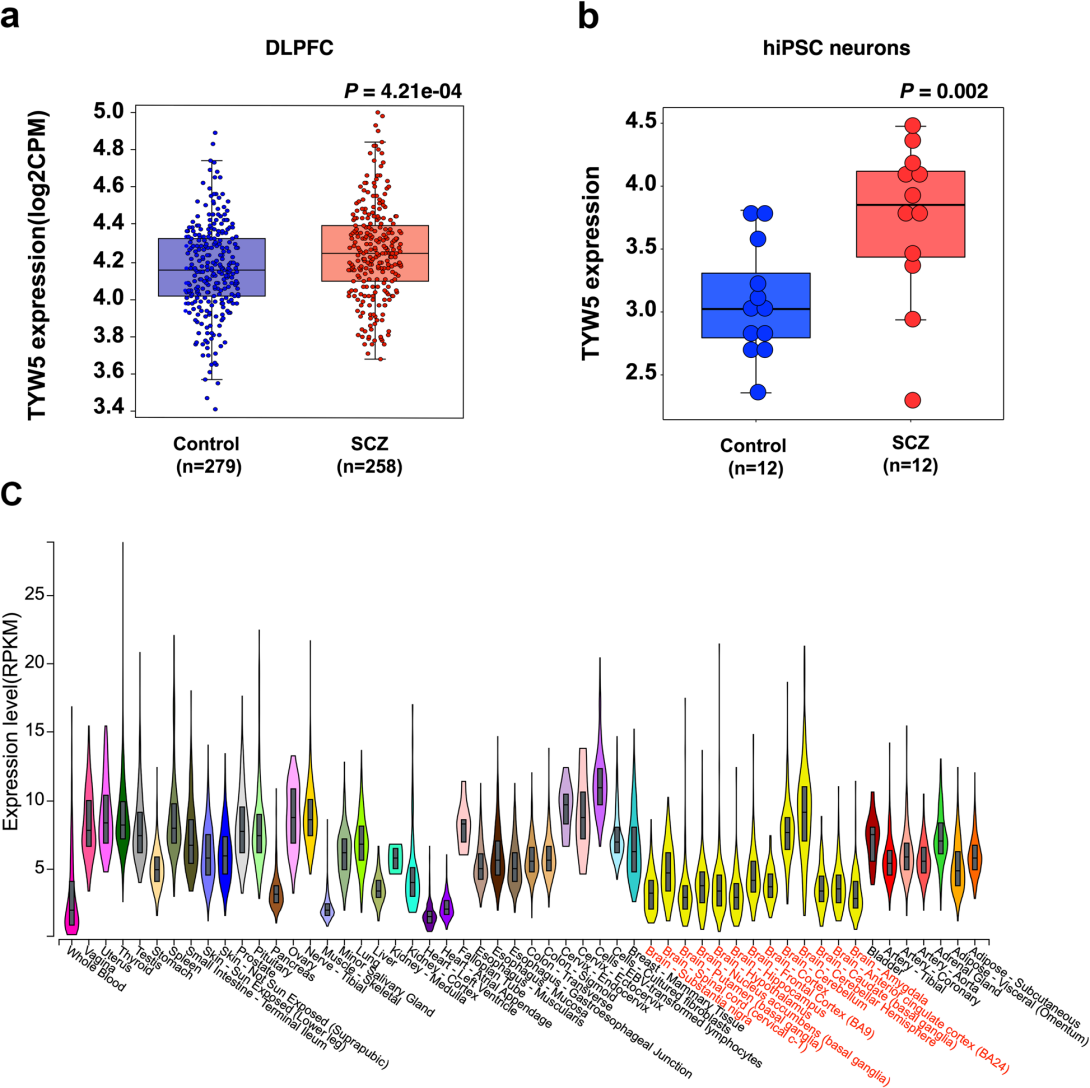
Significant upregulation of *TYW5* in brains of SCZ cases compared with controls. **a** *TYW5* was significantly upregulated in the hiPSC neurons of SCZ cases. **b** Compared with controls, *TYW5* was significantly upregulated in the dorsolateral prefrontal cortex (DLPFC) of SCZ cases. Student’s *t* test was used to compare if the difference was significant (*P* < 0.05, corrected). **c** *TYW5* is widely expressed in diverse human tissues. RNA sequencing-based expression data from GTEx was used to explore *TYW5* expression. *TYW5* was abundantly expressed in different human tissues, with the highest expression level in EBV-transformed lymphocytes

As previous studies showed that the dorsolateral prefrontal cortex (DLPFC) might have pivotal roles in SCZ, we compared the expression level of the significant genes (i.e., genes listed in Table S2) in the DLPFC of SCZ cases and controls using the expression data from Fromer et al. [22]. Among the genes listed in Table S1, only the *TYW5* gene showed a significant difference (*P* < 0.05, FDR corrected) in the DLPFC of SCZ cases compared with controls (Fig. 1a and Additional file 1: Table S2). Of note, *TYW5* was significantly upregulated in the DLPFC of SCZ cases compared with controls (*P* =4.21 × 10^−4^).

Furthermore, we examined changes in *TYW5* expression in neurons derived from iPSCs of SCZ patients and controls. Interestingly, the diagnostic analysis found that expression of *TYW5* was higher in neurons induced from iPSCs SCZ patients than healthy controls (SCZ cINs versus control cINs, *P*-value = 0.002; Fig. 1b), providing further evidence for the potential involvement of *TYW5* in SCZ.

### TYW5 is widely expressed in diverse human tissues

We examined *TYW5* expression in diverse human tissues using data from the GTEx. We found that *TYW5* was widely expressed in different human tissues, with the highest expression level in EBV-transformed lymphocytes (Fig. 1c). This expression data implies that *TYW5* may have a role in different human tissues.

### Cell specificity and spatio-temporal expression pattern of TYW5 expression in the central nervous system

**Fig. 2:**
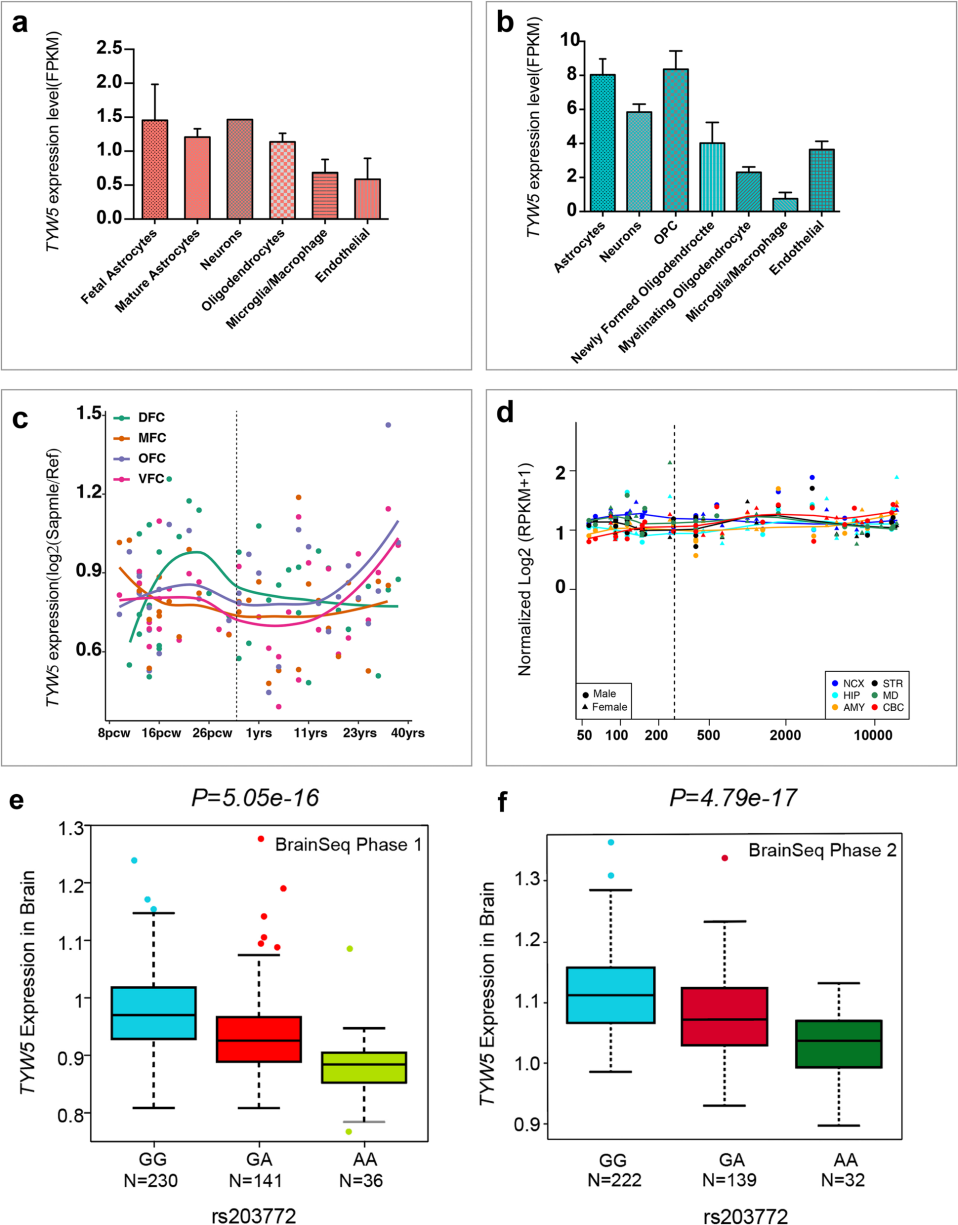
Expression of *TYW5* in different cell types of human and mouse brain and rs203772 may confer schizophrenia risk through regulating *TYW5* expression. **a** *TYW5* was expressed in different cell types (including neuron, microglia, oligodendrocytes, and astrocytes) of the human brain. **b** *TYW5* was abundantly expressed in different cell types of a mouse brain, with the relatively high expression level in astrocytes and orbitofrontal cortex (OFC) cells. **c, d** Expression of *TYW5* was relatively low at an early developmental stage. With the progress of development, *TYW5* expression was gradually increased and peaked at the adult stage. **c** Expression data from the BrainSpan was used for plotting. **d** Expression data from the PsychENCODE was used for plotting. **e** Association of rs203772 with *TYW5* expression in the 175 schizophrenia patients and 237 unaffected controls from DLPFC in BrainSeq Phase 1 dataset. **f** Association of rs203772 with *TYW5* expression in the DLPFC from 397 individuals in BrainSeq Phase 2 dataset

We further explored the expression of *TYW5* in different cell types (including astrocytes, neurons, oligodendrocytes, and microglia cells) of the central nervous system. Of note, we found that *TYW5* has the highest expression level in neurons and astrocyte cells of the human brain (Fig. 2a). To compare if *TYW5* has similar expression patterns in different cell types of human and mouse brains, we examined *TYW5* expression in different cell types of the mouse brain. We found that *TYW5* was expressed at higher levels in different cell types in the mouse brain compared to lower levels in different cell types in the human brain (Fig. 2b).

We further explored the expression pattern of *TYW5* in the developing and adult human brain using the expression data from the BrainSpan and PsychENCODE. We found that the expression of *TYW5* in the cortex was relatively low at an early developmental stage and gradually increased with the progress of development, and peaked at the adult stage (Fig. 2c). The expression level of T*YW5* was relatively stable among different brain regions (Fig. 2d). This temporal expression pattern suggests that *TYW5* may have different roles during brain development.

### rs203772 is associated with TYW5 mRNA expression

Although the samples in BrainSeq Phase 1 and 2 were partially overlapped, both datasets were included considering the different RNA-seq methods. We examined the association between rs203772 and TYW5 expression using data from BrainSeq Phase 1 and Phase 2. Interestingly, the SCZ risk SNP rs203772 was associated with TYW5 mRNA expression in human DLPFC tissues of 412 subjects (two-tailed *P* = 5.05 × 10^−16^) in BrainSeq Phase 1 dataset, with the risk allele (G) associated with higher TYW5 mRNA levels. In addition, we found a similar association trend in the DLPFC from 397 individuals in BrainSeq Phase 2 dataset (two-tailed *p* = 4.79 × 10^−17^, Fig. 2e, f).

### TYW5 expression-associated SNP (rs203772) showed a significant association with brain gray matter

**Fig. 3:**
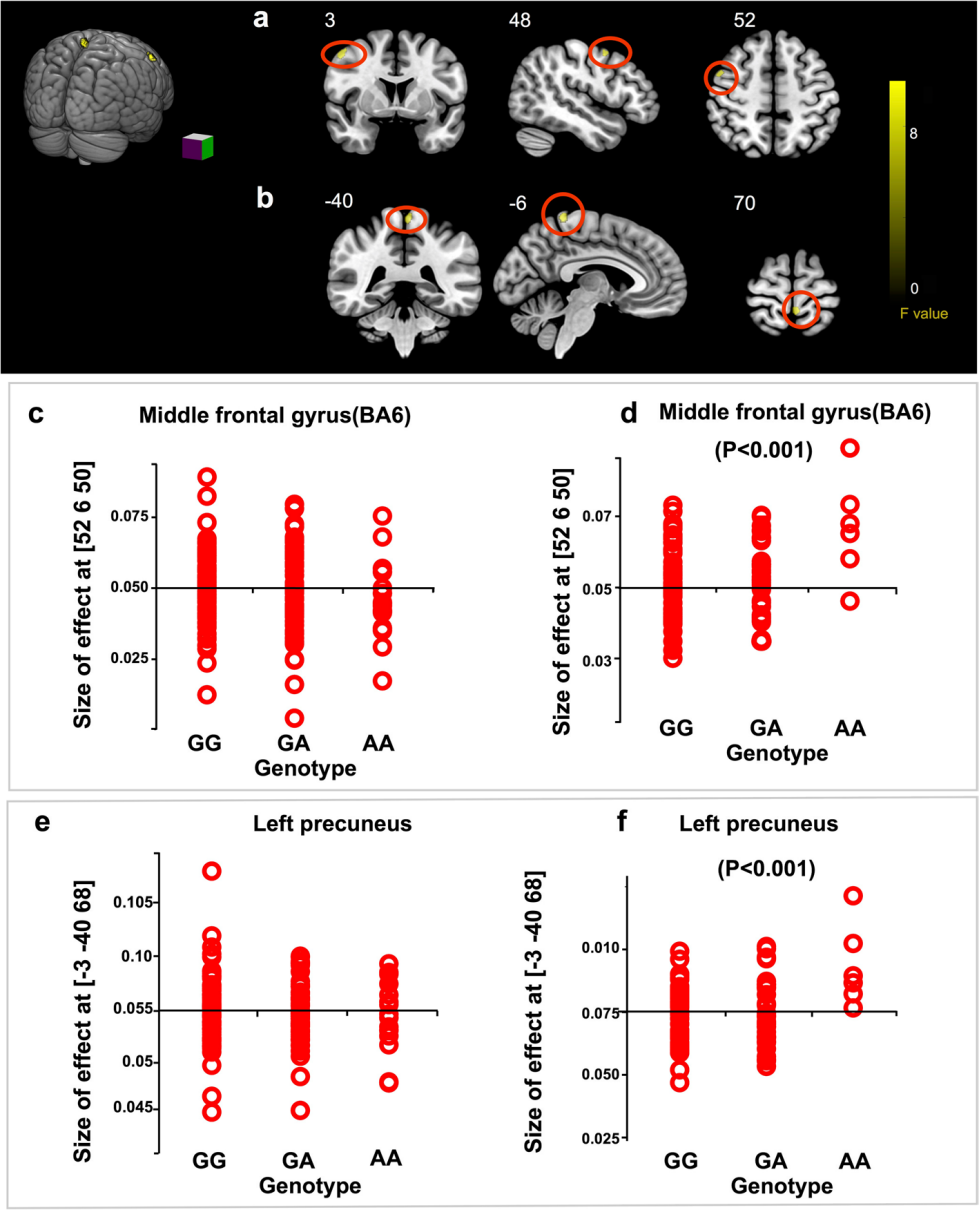
Effect of the risk single-nucleotide polymorphism (SNP) rs203772 on brain gray matter structure. The gray matter of right middle frontal gyrus (**a**) and left precuneus (**b**) in structural MRI. Carriers of the risk allele (G) of rs203772 in SCZ exhibit significantly decreased allele-dosage-dependent gray matter volume in the right middle frontal gyrus (**d**) and left precuneus (**f**), whereas the opposite trend of the right middle frontal gyrus (**c**) and left precuneus (**e**) was observed in healthy controls. Each red dot represents size of the effect in one subject and reflects gray matter volume

Previous studies have revealed that SCZ showed a significant genetic correlation with gray matter [18], an important risk factor for SCZ. Our Sherlock integrative analysis indicated that rs203772 was significantly associated with SCZ and *TYW5* expression simultaneously (Table 1), suggesting that SNP rs203772 may confer risk of SCZ through affecting *TYW5* expression. Considering the significant genetic correlation between SCZ and brain gray matter [52–54], we hypothesized that rs203772 might also be associated with gray matter abnormalities. We revealed a significant “diagnosis by genotype” interaction involving the right middle frontal gyrus and left precuneus. We found that patients who carried GG genotype had significantly lower right middle frontal gyrus and left precuneus volume compared to CC carriers. On the contrary, the controls showed a reverse pattern (Fig. 3). The association between rs203772 and abnormal gray matter provided additional evidence supporting rs203772 may represent a promising risk SNP for SCZ and abnormal brain structure.

## Discussion

Considering that most of the identified risk variants are located in the noncoding region, it is likely that these risk variants impart SCZ risk through altering gene expression. As the GWAS findings alone cannot predict whether the discovered SCZ risk variants have functional repercussions, a statistical method to combine data from all disease associations and independent expression QTL data is required [55]. In the present study, we utilized the largest GWAS of SCZ (PGC EAS+EUR) to date and conducted the genome-wide integrative analyses through combining brain eQTL, followed by independent replications in differential expression analysis in DLPFC and hiPSC neurons. Through this stepwise analysis, we found that *TYW5* is a new risk gene for SCZ. Besides, the risk allele of rs203772 (which predicts higher *TYW5* mRNA expression in the DLPFC) was also associated with the SCZ-relevant middle frontal gyrus and precuneus volumes in first-episode untreated samples. Our independent integrative analyses results provide convergent evidence to support the potential role of *TYW5* in SCZ.

*TYW5* is an essential tRNA hydroxylase, and previous studies have found that tRNA alteration defects are linked to many neurodevelopmental disorders [25, 56]. Accumulating evidence shows that *TYW5* is one of the best replicated SCZ risk genes [15, 57–61]. However, due to the high level of linkage disequilibrium, it is challenging to precisely locate the disease-causing gene in a small sample [15, 30]. In our study, the rs203772 in *TYW5* showed strong association with susceptibility to SCZ (*P* = 1.83 × 10^−8^), supporting that it is a true risk gene for SCZ. In addition, *TYW5* may also be involved in the genetic susceptibility of mental illness that shares the common risk factor with SCZ [62, 63], such as neurodevelopment-related disorders autism spectrum disorder [63] and bipolar disorder [62]. These lines of evidence provide convergent evidence to support that *TYW5* may represent an authentic susceptibility gene for SCZ [64]. In order to explore whether the *TYW5* identified by Sherlock integrative analysis were dysregulated in patients with SCZ, we also examined the brain expression level of *TYW5* in SCZ cases. We found that *TYW5* was significantly upregulated in DLPFC in patients with schizophrenia, which also suggests that *TYW5* may act as a potential therapeutic target of SCZ.

Although the pathophysiology of neuronal cell-specific damage in SCZ remains unclear, cortical neuronal abnormalities in SCZ have received extensive attention [65, 66]. The potential effects of *TYW5* on neuronal or synaptic function remain unclear [62, 67]. However, *TYW5* protein was likely expressed in cortical neurons during the process of synapse formation [67]. Our study found that *TYW5* is expressed in a variety of cell types in the human cerebral cortex [68], with the highest expression level in neurons and astrocytes. Our results indicate that *TYW5*–associated tRNA lesions in neurons and astrocytes may be one of the potential pathological changes of SCZ [69–71]. Next, we explored the differential expression of *TYW5* in induced pluripotent stem cells (iPSCs) and cortical interneurons in SCZ patients and healthy controls. We found that *TYW5* is significantly increased in cortical interneurons [72, 73], which is consistent with the dysregulated pattern in the DLPFC, suggesting that *TYW5* plays an important regulatory role in the development of cortical neuronal in SCZ [74]. In addition, our study also provides an opportunity to study the specific role of *TYW5* in the development of neural stem cells [75, 76].

Earlier studies have revealed abnormal neuronal differentiation, reduced synapse density, and abnormal expression of synaptic markers in the frontal lobe of SCZ patients [77]. Besides, in vitro and in vivo studies also discovered that genetic risk factors of SCZ usually result in disruption of synaptic morphology and function as well as brain circuits that are essential for positive symptoms and cognition, and thereby eventually lead to the onset of SCZ [78, 79]. Therefore, it is widely accepted that genetic factor in frontal dysfunctions play pivotal roles in the pathogenesis of SCZ [80]. The recent SCZ GWAS also supports this view, because genes involved in frontal eQTL and differential gene expression have been repeatedly emphasized [22, 81]. Our results indicate that the risk allele of rs203772 was associated with higher *TYW5* expression in the DLPFC. These results suggested the idea that the SCZ GWAS locus near rs203772 may confer risk of SCZ by regulating the expression level of the *TYW5* gene in frontal lobe brain tissue [82]. Furthermore, for the first time, by using neuroimaging results obtained from human subjects and ruled out the influence of drug confounding factors, we explored the genetic effects of *TYW5* on the entire brain gray matter with data-driven strategy. We found that risk allele of rs203772 (G) was associated with two special frontal sub-regions: the right middle frontal gyrus and left precuneus gray matter in first-episode schizophrenia. One of the possible functional mechanisms is that *TYW5* acts as a downstream regulator of the iron distribution pathway during normal and oncogenic neurodevelopment and may regulate the dopamine transporter by regulating the distribution of iron in the frontal lobe [83, 84]. From the perspective of neurodevelopmental function, *TYW5* may affect the development and function of the prefrontal cortex involved in the abnormal cognitive process of SCZ [15, 30, 85]. In addition, *TYW5* participates in mitochondrial biogenesis through interaction with methionyl-tRNA synthase 2, which may also be related to mitochondrial abnormalities related to frontal lobe development in the pathogenesis of SCZ [86]. Although the exact function of *TYW5* in this brain function is not yet clear, more functional studies are still urgently needed to gain insights on whether and how it affects brain circuits and behavior in disease-specific ways [87–89].

In addition to identify *TYW5* as a SCZ risk gene, other evidence also support that *TYW5* may play important roles in the central nervous system. Recent studies have also shown expression dysregulation of *TYW5* in cancer [90], including testicular germ cell tumors [91]. Also, studies have shown that *TYW5* regulates migration, invasion, and tumor cell proliferation [92]. These studies demonstrated the essential role of *TYW5* in cancer. Fascinatingly, schizophrenia has been reported to be a risk factor in cancer prognosis [93]. Also, earlier research suggested that SCZ prevalence was higher relative to cancer patients’ general population [94, 95].

Our study has several advantages. First, we used different integrative methods (by integrating eQTL and GWAS data) to discover and verify *TYW5* as a potential SCZ risk gene. In addition, protein integrative analysis using a large and comprehensive human proteome [96] and summary statistics from the most recent SCZ GWAS supported *TYW5* as a SCZ potential therapeutic targets. Finally, we found that rs203772 is also associated with gray matter abnormalities of the right middle frontal gyrus and left precuneus. The whole-brain imaging approach used in this work prevents empirical pre-selection of brain areas, and the first-episode, treatment-naive individuals avoid pharmaceutical confounding effects [97].

While this study offers some interesting observations, it should be noted that the present evidence is limited, and we interpret the results cautiously. First of all, due to the complexity of linkage disequilibrium and gene regulation, the causal (or functional) variants that regulate the expression of *TYW5* and the exact regulatory mechanism remain elusive. Second, different eQTL datasets might offer different results and refined single-cell expression data will promote the identified risk gene’s authenticity [98]. Third, our Sherlock analysis identified multiple genes whose expression disruption could play a role in SCZ; however, in this study we only focused on the top significant *TYW5* after multiple corrections. More work is required to illuminate whether other risk genes identified by Sherlock integrative analysis also have a role in SCZ. Furthermore, we would replicate the association between the rs203772 genotypes and grey matter abnormalities in an independent patient cohort in future research. Finally, *TYW5* expression modulation in related (eventually patient) cell lines or animal models will provide further evidence for the potential role of *TYW5* in schizophrenia.

## Conclusions

In summary, our comprehensive study identifies *TYW5* as a new SCZ risk gene whose expression level may contribute to SCZ risk. Our study links SCZ risk variants to specific genes and provides a possible mechanistic explanation between genetic variation and SCZ susceptibility. This study links some of the risk variants from the largest GWAS of SCZ to specific genes. It provides a framework to investigate how genetic variants contribute to SCZ risk by modulating gene expression and provides a starting point to dissect the possible role of the identified genes in the brain morphology of SCZ.

## Supplementary Information

**Additional file 1: Figure S1.** Workflow of integrative analyses in this study. **Table S1.** Significant dysregulation of TYW5 in DLPFC of SCZ cases compared with controls. **Table S2.** Comparative Profile of rs203772 genotype distribution between first-episode untreated SCZ patients & controls.

## Data Availability

All data relevant to the study are included in the article or uploaded as online supplementary information. The data generated in this study and codes associated with the current submission will be available from the corresponding author on reasonable request.

## Abbreviations

EAS: East Asian ancestry
eQTL: Expression quantitative trait locus
EUR: European ancestry
GWAS: Genome-wide association studies
iPSCs: Induced pluripotent stem cells
pQTL: Protein quantitative trait locus
SCZ: Schizophrenia
SMR: Summary data-based Mendelian Randomization
SNPs: Single-nucleotide polymorphisms
TWAS: Transcriptome-wide association study
