# Supplementary material for: Comprehensive and integrative analyses identify TYW5 as a schizophrenia risk gene
Source: BMC Med. 2022 May 9;20:169. doi: 10.1186/s12916-022-02363-8 (PMC9082878; doi:10.1186/s12916-022-02363-8)
Supplement: Supplementary file 1 — Additional file 1: Figure S1. Workflow of integrative analyses in this study. Table S1. Significant dysregulation of TYW5 in DLPFC of SCZ cases compared with controls. Table S2. Comparative Profile of rs203772 genotype distribution between first-episode untreated SCZ patients & controls. [file 12916_2022_2363_MOESM1_ESM.docx]

**Supplementary Material for**

**Comprehensive and integrative analyses identify *TYW5* as a schizophrenia risk gene**

Chengcheng Zhang, Xiaojing Li, Liansheng Zhao, Rong Liang, Wei Deng, Wanjun Guo, Qiang Wang, Xun Hu, Xiangdong Du, Pak Chung Sham, Xiongjian Luo, Tao Li

**Supplementary tables and figures**

**Figure S1:** Workflow of integrative analyses in this study. We begin by combining GTEx eQTL data with PGC EAS+EUR GWAS data using Sherlock. We utilized SMR to link the eQTL data from GTEx brain tissue and the pQTL data from ROSMAP/Banner brain tissue with the PGC EAS+EUR GWAS data for additional verification, based on Sherlock's findings. Following that, we mapped *TYW5* mRNA expression patterns in healthy controls and schizophrenics using data from the CommonMind Consortium and iPSCs. We looked at the relationship between rs203772 and *TYW5* expression using BrainSeq phase I and phase II datasets. Finally, we used structural MRI to look at the link between rs203772 and gray matter anomalies in first-dose schizophrenia.

**Table S1:** Significant dysregulation of TYW5 in DLPFC of SCZ cases compared with controls.

**Table S2:** Comparative Profile of rs203772 genotype distribution between first-episode untreated SCZ patients & controls.

**Figure S1. Workflow of integrative analyses in this study.** We begin by combining GTEx eQTL data with PGC EAS+EUR GWAS data using Sherlock. We utilized SMR to link the eQTL data from GTEx brain tissue and the pQTL data from ROSMAP/Banner brain tissue with the PGC EAS+EUR GWAS data for additional verification, based on Sherlock's findings. Following that, we mapped *TYW5* mRNA expression patterns in healthy controls and schizophrenics using data from the CommonMind Consortium and iPSCs. We looked at the relationship between rs203772 and *TYW5* expression using BrainSeq phase I and phase II datasets. Finally, we used structural MRI to look at the link between rs203772 and gray matter anomalies in first-dose schizophrenia.

| Table S1. Comparative Profile of rs203772 genotype distribution between first-episode untreated SCZ patients & controls. | | | | |
| --- | --- | --- | --- | --- |
| rs203772 | Frequency [number of samples] | | x^2^ | *P* |
|  | Patients [86] | Controls [152] |  |  |
| GG | 0.55（47） | 0.52（79） | 0.83 | 0.66 |
| GA | 0.38（33） | 0.37（57） |  |  |
| AA | 0.07（6） | 0.11（16） |  |  |

| Table S2. Significant dysregulation of TYW5 in DLPFC of SCZ cases compared with controls. | | | | | | | |
| --- | --- | --- | --- | --- | --- | --- | --- |
| **Gene** | **Genome Position** | **logFC** | **Avg Expression** | **T Statistic** | ***P*-value** | **FDR^a^** | **B coefficient** |
| TYW5 | chr2:200794698-200820459 | 0.087 | 4.641 | 3.547 | 4.21E-04 | 2.65E-02 | -0.227 |
| C4A | chr6:31949801-31970458 | 0.19 | 1.393 | 1.878 | 6.09E-02 | 2.58E-01 | -4.165 |
| BAG6 | chr6:31606805-31620482 | -0.021 | 5.95 | -0.849 | 3.96E-01 | 6.54E-01 | -5.948 |
| PCDHA10 | chr5:140235595-140391929 | -0.034 | 1.885 | -0.388 | 6.98E-01 | 8.55E-01 | -5.749 |
| BTN3A2 | chr6:26365387-26378546 | 0.129 | 2.412 | 2.229 | 2.62E-02 | 1.70E-01 | -3.61 |
| MAPK3 | chr16:30125426-30134827 | -0.046 | 5.088 | -1.173 | 2.41E-01 | 5.10E-01 | -5.585 |
| PCDHA9 | chr5:140227048-140391929 | -0.003 | 2.675 | -0.039 | 9.69E-01 | 9.86E-01 | -5.944 |
| **^a^** corrected p<0.05 | | | | | | | |
